# Role of cohesion in the flow of active particles through bottlenecks

**DOI:** 10.1038/s41598-022-15577-w

**Published:** 2022-07-07

**Authors:** Timo Knippenberg, Anton Lüders, Celia Lozano, Peter Nielaba, Clemens Bechinger

**Affiliations:** 1 Fachbereich Physik, Universität Konstanz, 78457 Constance, Germany; 2 Bosonit, AI Department, 26006 La Rioja, Spain

**Keywords:** Applied physics, Statistical physics, thermodynamics and nonlinear dynamics

## Abstract

We experimentally and numerically study the flow of programmable active particles (APs) with tunable cohesion strength through geometric constrictions. Similar to purely repulsive granular systems, we observe an exponential distribution of burst sizes and power-law-distributed clogging durations. Upon increasing cohesion between APs, we find a rather abrupt transition from an arch-dominated clogging regime to a cohesion-dominated regime where droplets form at the aperture of the bottleneck. In the arch-dominated regime the flow-rate only weakly depends on the cohesion strength. This suggests that cohesion must not necessarily decrease the group’s efficiency passing through geometric constrictions or pores. Such behavior is explained by “slippery” particle bonds which avoids the formation of a rigid particle network and thus prevents clogging. Overall, our results confirm the general applicability of the statistical framework of intermittent flow through bottlenecks developed for granular materials also in case of active microswimmers whose behavior is more complex than that of Brownian particles but which mimic the behavior of living systems.

## Introduction

When particles are forced through a sufficiently narrow geometric constriction, their flow becomes unsteady due to the development of temporary clogs, e.g. arches, which strongly perturb their free motion. Such behavior has been studied in great detail, e.g. for granular matter driven through funnels^1–4^ or colloidal particles flowing through geometric constraints^5–11^. Independent of the specific system, empirically, one finds an intermittent particle current which is governed by exponentially distributed burst sizes and a power-law dependence of the clogging times^1^. In addition to particles which are driven through constrictions by external forces or fluid flows, clogging is also observed in self-propelling systems, e.g. sheep herds, pedestrian crowds or active grains^12–17^. Despite considerable differences compared to externally driven systems, their intermittent clogging behaviour is very similar^1^. However, the effect of cohesion, which is often dominating the behavior of group-forming living systems, has not been investigated so far.

In our study, we experimentally investigate a system of active colloidal particles (APs) which are propelled through a two-dimensional (2D) funnel and whose mutual interactions can be precisely controlled via an optical feedback-loop. In our specific case, we considered APs whose interactions are dominated by cohesion and alignment, being motivated by social rules governing the behaviors in many living collective systems^18–20^. Similar to other studies without cohesive interactions, we find an exponential burst distribution and an algebraic decay in the clogging time distribution. Remarkably, we find that cohesion has, however, only a very weak influence on the particle flow, up to a certain threshold. Such counter-intuitive behavior is caused by short-ranged AP repulsions which leads to a rather high mobility of group members. Our results are corroborated by 2D Brownian dynamics (BD) simulations using a minimal model of active Brownian particles with an effective temperature. Our results confirm the robustness of the mentioned framework even in presence of complex and non-reciprocal interactions.

## Experimental methods

Our experiments were performed with a suspension of programmable APs using a feed-back loop where the corresponding information and steering signals, determined by social interaction rules, are instantaneously computed externally and then inserted into the system. This type of approach has already been demonstrated to reproduce collective behaviors of swarms and swirls which are often observed in living systems^21,22^. In our work, groups of responsive APs are made from transparent silica particles with diameter $\sigma$ = 6.16 µm which are coated on one side with an 80 nm thick, light absorbing carbon cap (SI). They are suspended in a critical mixture of water and propylene glycol n-propyl ether (PnP) (40%m) contained in a thin sample cell whose temperature is kept constant and below the fluid’s lower demixing temperature *T*_*c*_ = 31.9 °C^23^. When an AP is illuminated with a focused laser beam, its cap heats up and induces a local demixing which leads to a fluid flow around the AP^24,25^. This results in a quasi-2D self-propulsive, i.e. active, particle motion, with velocity $\overrightarrow{u}=v \widehat{\overrightarrow{u}}$. Here, $v$ corresponds to the magnitude and $\widehat{\overrightarrow{u}}$ to the direction of motion which is opposite to the carbon cap. The 2D restriction of the APs orientational and translational motion is due to gravity and hydrodynamic effects^26^. To impose well-controlled social interaction rules, their velocity (magnitude and direction) is controlled individually. This is achieved by scanning the illuminating laser beam with an acousto-optic deflector (AOD) rapidly across all APs and by independently adjusting the intensity and laser focus position relative to each cap centre^22^ (SI). Via feedback-control, we are able to apply effective self-propelling forces and torques to the APs, which enables them to stop, move forward or turn to the left and right depending on the chosen interaction rule. Despite similarities with a simulation, it should be noted that in our experiments, the APs interact with a true physical environment which leads to additional, e.g. steric, hydrodynamic and phoretic, interactions which are frequently neglected in numerical studies.

### Interaction rules

To create a cohesive group of $N$ APs, each particle must follow a certain interaction rule according to which it adjusts its direction of motion relative to its surrounding.

Our specific choice is largely motivated by previous numerical studies which yields a collective and cohesive motion of APs based on strictly local interaction rules^19,27^. Within this approach, the direction of motion $\widehat{\overrightarrow{{u}_{i}}}=\overrightarrow{{u}_{i}}/v$ of particle $i$ at position $\overrightarrow{{r}_{i}}$ depends on the orientation and distance to its neighbours ${r}_{ij} = | \overrightarrow{{r}_{j}} -\overrightarrow{{r}_{i}} |$ according to

1 $$\widehat{\overrightarrow{{u}_{i}}}=-{\left \langle \upvarepsilon \frac{\overrightarrow{{r}_{j}}\left(t\right)-\overrightarrow{{r}_{i}}\left(t\right)}{{r}_{ij}} \right \rangle }_{j,{r}_{ij}\le {r}_{\text{c}}}+\upbeta {\left \langle {f}_{ij}\left(\overrightarrow{{r}_{i}}\left(t\right),\overrightarrow{{r}_{j}}\left(t\right)\right)\frac{\overrightarrow{{r}_{j}}\left(t\right)-\overrightarrow{{r}_{i}}\left(t\right)}{{r}_{ij}} \right \rangle }_{j,{r}_{\text{c}}<{r}_{ij}\le {r}_{0}}+\upgamma \cdot {\overrightarrow{e}}_{\text{drive}}$$

Here, $\langle \cdot {\rangle }_{j, {r}_{ij}\le {r}_{\text{c}}}$ and $\langle \cdot {\rangle }_{j,{r}_{\text{c}}<{r}_{ij}\le {r}_{0}}$ denote the average over all neighboring particles with a distance of ${r}_{ij}\le {r}_{\text{c}}$ and ${r}_{\text{c}}<{r}_{ij}\le {r}_{0}$ respectively. The direction $\widehat{\overrightarrow{{u}_{i}}}$ is normalised before being applied to the particle.

The first two terms (adapted from^19^) characterise centre-to-centre interactions with neighbours, which are composed of a short-ranged repulsion scaled by $\varepsilon$, a long range attraction ${f}_{ij}$ with prefactor $\beta$, yielding a minimum at distance ${r}_{e}$.

2 $${f}_{ij}\left(\overrightarrow{{r}_{i}}, \overrightarrow{{r}_{j}}\right)=\left\{\begin{array}{ll}\frac{1}{4}\frac{{r}_{ij}-{r}_{\text{e}}}{{r}_{\text{a}}-{r}_{\text{e}}},& \quad {r}_{\text{c}}<{r}_{ij}\le {r}_{\text{a}}\\ 1, & \quad {r}_{\text{a}}<{r}_{ij}\le {r}_{0}\end{array}\right.$$

To steer the entire group towards an obstacle at a fixed position, we have added a global directional unit vector ${\overrightarrow{e}}_{\text{drive}}$ with length $\gamma$ to the motional direction of each particle. It should be noted that this vector also leads to a high degree of polarisation within the group, similar to a flock (see supplementary movie M1).

In the experiments and simulations presented below, we have chosen the AP velocity $v \approx$ $0.19 \space \frac{\mu m}{s}$, and set the other parameters to $\gamma =1$, ${r}_{\text{c}} = 1.6 \sigma , {r}_{\text{a}}=4.4 \sigma , {r}_{\text{e}} = 2.4 \sigma$ and $\varepsilon = 9000$. Note that as a result of viscous friction between the APs and the solvent instantaneous changes in $\widehat{\overrightarrow{{u}_{i}}}$ are not possible but only achieved with an angular rate $\omega \approx 1.5 \space^\circ /s$ in our experiments (for details, see^22^ and SI Fig. S1). In particular at small distances ${r}_{ij}\le {r}_{\text{c}}$, this renders the actual AP motion more complex than what is typically considered in numerical simulations with non-polar particles, e.g. by enabling close particle.

**Figure 1:**
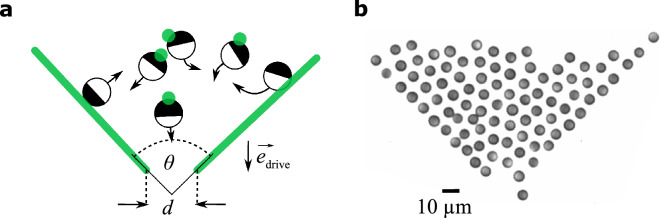
Experimental realisation. (**a**) Sketch of the experimental setup: Janus particles are located inside a bottleneck (width $d$ and opening angle $\theta$) set by a constant applied light gradient. The active motion is induced by targeting the APs with single laser spots (sketched as green circles). (**b**) Microscope image of the experimental implementation of a bottleneck with $\theta =90^\circ$, $d=2.3 \sigma$ and $\beta =0$.

For the realisation of a bottleneck, we have created a funnel-shaped light pattern composed of two lines at an angle $\theta$ and an aperture $d$ using the scanning laser beam as discussed above (and SI) and as sketched in Fig. 1a. When particles approach these lines, they will be reflected due to the corresponding phototactic response which leads to an effective torque opposite to the light gradient^25^. This leads to an effective barrier for the particles. Note that the barrier is modeled intransparent, meaning that if the connecting line of particle positions crosses the barrier, their contribution is ignored in the interaction rule between APs. Figure 1b shows a microscope image of a group of APs following the above introduced interaction rule in the presence of the bottleneck. As can be seen, the phototactic repulsion with the virtual walls is sufficient to provide an effective confinement for the system.

### Cohesion-dependent clogging behavior

**Figure 2:**
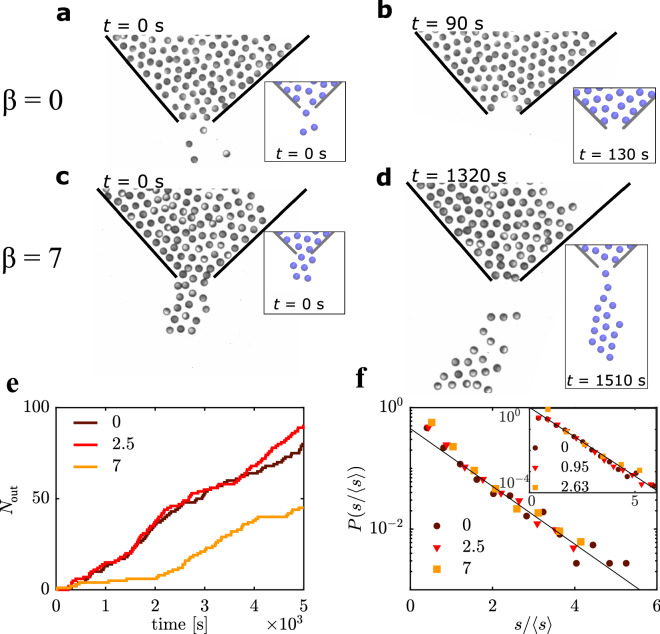
Clogging mechanisms. Snapshots of the bottleneck in the two different regimes: For $\beta = 0$ the particle flow (**a**) is interrupted by arches (**b**). For $\beta = 7$ , droplets (**c**) form and eventually detach (**d**). The insets show the correspondent situations as observed in numerical simulations at the same $\beta /{\beta }_{0}$. The images of the inset were created using Visual Molecular Dynamics (VMD)^28^. (**e**) Exemplary curves of the cumulate number of particles having passed the obstacle. The colours refer to values of $\beta$. (**f**) Probability distribution function of burst sizes $s$ normalised by the average burst size $\langle s\rangle$ for different values of $\beta$. Note the log-lin scale. The solid line is a fit $\propto {e}^{-1.1\cdot s/\langle s\rangle }$. The inset is the associated simulation data, where we find $P(s/\langle s\rangle )\propto {e}^{-1.5\cdot s/\langle s\rangle }$. For the calculation, we register the time points at which particles enter a registration-area of length $d$ and width $\sigma$, directly located at the aperture. In all figures, it is $\theta = 90^\circ$ and $d = 2.3 \sigma$.

Figure 2 shows snapshots at different times obtained from experiments for $\beta = 0$ (a, b) and $\beta = 7$ (c, d) and an aperture of *d*  = 2.3 $\sigma$.

For $\beta =0$, we observe the system to rapidly alternate between being clear (i.e. particles are able to pass the bottleneck) (Fig. 2a) and being clogged (Fig. 2b) (supplementary movie M2). The latter is caused by arch-like structures, similar to those previously reported for vibrated granular matter^29,30^ and passive colloids^7,31^. Arch-formation in dense particle systems in presence of an external force is a well-known phenomenon, which evolves due to particles blocking each other while competing for space^32^ and leads to an interruption of the flow. The corresponding cumulative number of particles ${N}_{\text{out}}$(*t*) having passed the bottleneck at time *t* is shown in Fig. 2e, featuring an intermittent flow. In the following, we call this behavior the arch-dominated regime.

With increasing $\beta$, the passage of APs through the bottleneck becomes qualitatively different. This is clearly seen in Fig. 2c where a “droplet” of APs is formed at the aperture which eventually detaches and moves along ${\overrightarrow{e}}_{\text{drive}}$ (Fig. 2d) (supplementary movies M3 and M4). Similar droplet formation has also been reported for wet granular materials when passing through confinements^33^. Compared to arches, which are less stable, the formation of droplets provides a much more robust clogging mechanism which is consistent with considerably larger clogging times between particle bursts in this cohesion-dominated regime (Fig. 2e). After being detached from the bottleneck, the droplets move with rather constant velocity, similar to droplets formed in binary microfluidic flows^34^ (supplementary movie M5). Within the range of $\beta$-values considered in this work, the droplets typically consisted of 20 to 50 APs.

When systematically increasing the cohesion in our experiments we find a transition between both regimes around ${\beta }_{0}\approx 4$. This has been determined in consideration of the clogging statistics as explained below. For easier comparability, we specify the cohesion in terms of normalised $\beta /{\beta }_{0}$ values in the following.

For further analysis, we captured the burst sizes s (i.e. the number of particles passing the bottleneck without a separating gap time larger than ${t}_{\mathrm{f}} =2 \frac{\sigma }{v} \approx 64$ s. As expected^32^, they are exponentially distributed $\propto {e}^{-1.1 s/\langle s\rangle }$ independently of $\beta$ (Fig. 2f) when normalised by the average burst size $\langle s\rangle$. In general, the above behavior is consistent with the assumption of a time-independent probability to form a blockage, as explained in theoretical studies^12^.

### Power law behavior and flow rate

**Figure 3:**
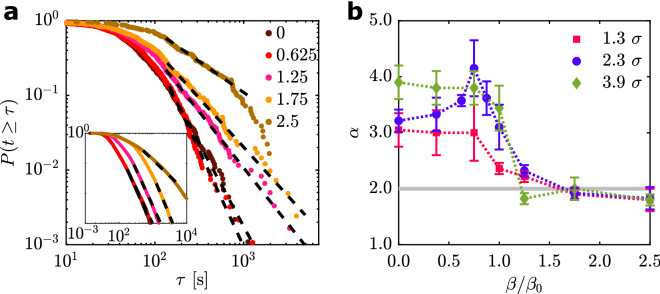
Cumulative distribution functions. (**a**) The complementary cumulative distribution functions $P(t \ge \tau )$ of time $\tau$ between subsequent particle registration events as obtained from experiments with $\theta =90^\circ$ and $d=2.3 \sigma$*.* Different colours refer to different values of $\beta /{\beta }_{0}$. The dashed lines are power-law fits, the start of the power law section and the corresponding exponent was obtained using the procedure proposed in^36^. The obtained exponents are for increasing $\beta$: $\alpha = 3.2, 3.6, 2.3, 1.9, 1.8$. The inset shows the corresponding simulation data with exponents: $\alpha = 3.5, 3.4, 3.5, 3.7, 2.1$. (**b**) Exponents as extracted from experimental CDF fits for bottlenecks with $\theta =90^\circ$ and varying $d$. Colours refer to values of $d$. For information about the determination of uncertainties, see SI.

To further quantify the intermittent particle flow through the bottleneck, we evaluated the complementary cumulative distribution function (CDF) $P(t \ge \tau )$ of the time lapses $\tau$ between the consecutive passage of APs. The results for $\theta = 90^\circ$ and $d=2.3 \sigma$ obtained from experiments for different cohesion strengths are shown in Fig. 3a. We observe an initial plateau followed by a power-law decay at larger $\tau$, i.e. $P(t \ge \tau )\propto {\tau }^{-\alpha +1}$ with $\alpha$ in the range of 2 to 4.5 depending on $\beta /{\beta }_{0}$. Such behavior is in good agreement with established results of sheep, humans, small robots and inanimate granular matter passing geometric confinements^8,16,17,30,35^ and thus demonstrates such power-law tails to be an universal feature independent of the type and size of passing particles. In general, a power-law tail with an exponent $\alpha \le 2$ corresponds to a divergence of $\langle \tau \rangle$, i.e. a permanent clogging for sufficiently large time scales and particle numbers^1^. Our data demonstrates values of the exponent in the vicinity of $\alpha \approx 2$ for $\beta /{\beta }_{0} > 1$ and for different *d* (Fig. 3b), consequently the system approaches diverging clogging times there. The decrease of $\alpha$ from values between 3 and 4.5 to $\alpha \approx 2$ coincides with the change from the arch-dominated to the cohesion-dominated regime at ${\beta }_{0}$ (Fig. 3b). Similar results have been also obtained for other values of $\theta$,* d*, and $\beta$ (SI Fig. S2).

In particular for strong cohesion $\beta /{\beta }_{0} > 1$, the probability of long clogging times increases as the exponent approaches $\alpha \approx 2$, which can lead to a poor convergence of the CDFs within our measurement time scales ${t}_{\text{meas}}\approx 4000 \space {\text{s}}$. Additionally it is less clear whether unique power-law tails in the CDF validly describe systems of APs featuring such high cohesion, as it is known from granular systems. This makes the interpretation of an exponent in the vicinity of $\alpha \approx 2$ more difficult. Therefore, in the following we will study the particle flow rate ${R}_{\mathrm{flow}}$ through the bottleneck which is based on the average time for the entire group to pass the bottleneck $\langle {t}_{\text{tot}}\rangle$. The choice of ${R}_{\mathrm{flow}}$ is largely motivated by recent experiments of flowing granular matter which demonstrated that such a measure also allows quantification of intermittent particle flow^30^ while converging faster than the CDF (SI). In addition, this quantity seems also more appropriate to characterise the effectiveness of how a finite cohesive group is able to pass an obstacle. For the calculation of ${R}_{\mathrm{flow}}$, we define the average time $\left\langle {\tau } \right\rangle_{{{\text{meas}}}} ~ = \mathop \smallint \limits_{0}^{{t_{{{\text{meas}}}} }} ~\tau ~p_{{{\text{exp}}}} ~\left( \tau \right)~{\text{d}}\tau$ for two consecutive APs to pass the aperture as obtained in the experiments with a finite measurement time scale ${t}_{\text{meas}}$. Here, ${p}_{\mathrm{exp}}\left(\tau \right)$ is the corresponding probability density function of $\tau$. Note that this integral does not converge for $\alpha \le 2$ and ${t}_{\text{meas}}\to \infty$ in case of a power-law tail of the corresponding CDF^36^. Thus, the average ${\langle \tau \rangle }_{\text{meas}}$ depends on ${t}_{\text{meas}}$.

**Figure 4:**
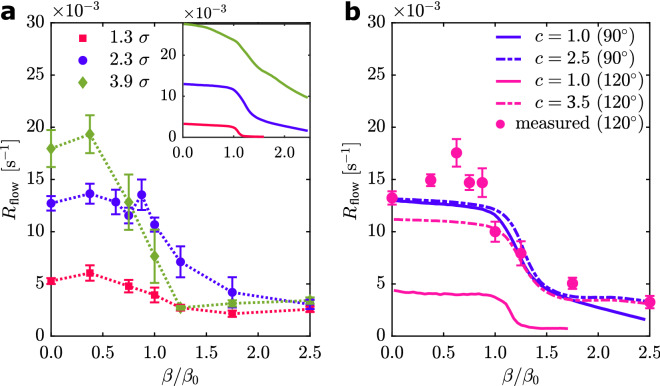
Flow rate. (**a**) Flow rate ${R}_{\text{flow}}$ at a bottleneck with $\theta =90^\circ$ and varying $d$. Experimental (data points connected by dashed lines) and simulated (inset) data. The error bars correspond to the standard deviation of mean of the single measurements. (**b**) Simulated flow rate at a bottleneck with $d = 2.3 \sigma$ and $\theta =90^\circ {\text{or}}$ $\theta =120^\circ$. The prefactor of the simulated effective temperature c is varied (dashed and straight lines). The datapoints are the experimental results for a bottleneck with $d = 2.3 \sigma$ and $\theta =120^\circ$. The error bars correspond to the standard deviation of mean of the single measurements.

From $\langle {t}_{{\text{to}}{\text{t}}}\rangle$ and ${\langle \tau \rangle }_{\text{meas}}$ one obtains the particle flow rate

3 $${R}_{\text{flow}} =\frac{N}{\langle {t}_{\text{tot}}\rangle }= \frac{N}{\left(N-1\right){\langle \tau \rangle }_{\mathrm{meas}} } \propto \frac{1}{{\langle \tau \rangle }_{\mathrm{meas}} } ,$$

which is shown as symbols as a function of $\beta$ and for different bottleneck apertures d in Fig. 4a. As expected, the flow rate increases with increasing $d$*.* Interestingly, the flow rate first remains rather constant in the range $\beta < {\beta }_{0}$ and rapidly drops at $\beta \approx {\beta }_{0}$, independent of $d$. This demonstrates that cohesion must not necessarily be detrimental to the efficiency of groups passing through bottlenecks. This at first glance unintuitive behavior has also been partially observed in dense colloidal systems of attractive Brownian, i.e., passive particles transported within a laminar fluid flow through a constriction^31,37^.

In those experiments, particle attractions even on the order of 10 ${k}_{\text{B}}T$ have been demonstrated to not necessarily lead to clogging. This has been explained by the presence of “slippery” particle bonds which avoids the formation of a rigid particle network and thus prevents clogging. The absence of such rigid networks is in agreement with numerical simulations of APs following similar interaction rules as used by us, which demonstrate the presence of a fluid-like AP behavior up to rather large values of the cohesion parameter^27^.

In case of attractive Brownian particles, the flow eventually approaches zero for large attractions, i.e. the system enters a permanently clogged state. Instead, in our experiments, the measured flow rate remains finite even at large cohesion as shown in Fig. 4a, even in the $\beta$ range where we obtained exponents in the vicinity of $\alpha \approx 2$. Such finite flow rates at $\beta /{\beta }_{0} > 1$ are also demonstrated by the fact that in the cohesion dominated regime, the emerging droplets at the aperture seem to eventually detach (cf. Fig. 2c,d) enabling AP passage. The described behaviour has been observed for various values of $\theta$, $d$ and $\beta$. Note that while our experiments suggest that all droplets eventually detach so that particles can pass the bottleneck, statements regarding a permanent clogging for ${t}_{\text{meas}}\to \infty$ and an infinite number of APs (as indicated by $\alpha$) are not possible with our data due to our finite measurement time scale. The opening angle has only a marginal influence on the experimental results. As an aside, note that an estimation of how the measurement time affects the uncertainty in determining the flow rate is presented in the SI.

**Figure 5:**
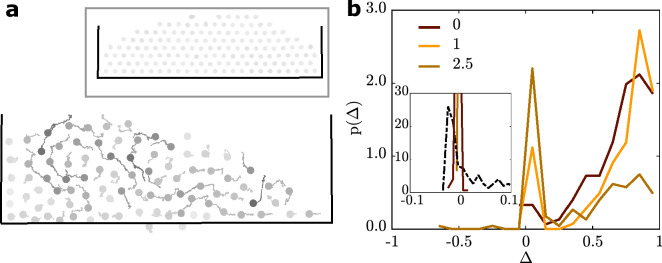
Particle dynamics. (**a**) Exemplary particle trajectories inside a rectangular confinement at $\beta /{\beta }_{0}=1.25$ in the experiment and in the simulation (inset) captured within $135 \space {\text{s}}$. The grayscale is proportional to the trajectory length, same scale for experiment and simulation. (**b**) Probability density function p $(\Delta )$ for particles in a confined group for experiment and simulation (inset). The line colours refer to values of $\beta /{\beta }_{0}.$ The dashed line in the inset corresponds to an effective temperature of $c= 2.5$ and $\beta /{\beta }_{0} = 0$, while the continuous lines refer to systems with $c= 1$. See SI (Fig. S3) for the full measured dataset.

To get more insight into the structure of such non-rigid networks, which form even at large values of $\beta$, we have studied their positional fluctuations within the group. To avoid a centre-of-mass motion of the group, we have studied their trajectories within a closed confinement (supplementary movie M6). To ensure that the APs have reached a steady state, we waited 2500 s after initialising the experiment before starting to record the data. Figure 5a shows an example for cohesion $\beta /{\beta }_{0}= 1.25$ where the particles still exhibit strong relative motion.

To quantify such relative particle displacements, we use the order parameter $\Delta$^27^, which measures the mean squared displacements of initially neighboring particles within a time interval $\chi$

4 $$\Delta ={\left \langle \frac{1}{{n}_{i}}\cdot {\sum }_{j=1}^{{n}_{i}}\left(1-\frac{{r}_{ij}^{2}\left(t\right)}{{r}_{ij}^{2}\left(t+\chi\right)}\right) \right \rangle }_{i} .$$

Here, the index $j$ corresponds to the neighboring particles (${r}_{ij}(t)\le {r}_{\text{e}}$) of particle $i$ and ${n}_{i}$ the corresponding coordination number. We choose $\chi =1800$ s, which is large enough to allow APs to sample their configurational space relative to their next neighbours. Such an order parameter is $\Delta \approx 0$ for a solid-like phase, but $\Delta \approx 1$ for a liquid phase in which APs are able to switch next neighbours^27^. Figure 5b shows the measured probability distribution function p($\Delta$) of confined AP groups at varying $\beta /{\beta }_{0}$. Clearly, the maximum of the distribution is at $\Delta >0$ indicating a liquid-like behavior for $\beta /{\beta }_{0 }= 0$. Even though a growing peak at $\Delta \approx 0$ appears with increasing cohesion, there is a remaining contribution at $\Delta >0$ even for the highest values of $\beta /{\beta }_{0}$. Qualitatively, this is caused by the above-mentioned AP reorientation mechanism which makes them to turn away from each other when their centre-to-centre distance falls below ${r}_{\text{c}}$. As a result of this particle-collision avoidance motion, positional AP fluctuations increase with increasing cohesion and this explains why the system remains fluid-like even at large values of $\beta$.

### Simulations

To consolidate our insights, we corroborate our experimental results with 2D Brownian dynamics (BD) simulations employing a minimal model of active Brownian particles. Within this generic model, hydrodynamic interactions are neglected. Otherwise, the particles are interacting with equivalent interaction rules as used in the experiments, where the mutual particle repulsion for ${r}_{ij}\le {r}_{\text{c}}$ is modeled after the well-known Weeks-Chandler-Andersen (WCA) potential^38^. The walls forming the funnel are implemented as soft wall barriers also using the WCA potential. Due to natural variations in the preparation process of APs, their velocity is not exactly constant but somewhat distributed. We modeled this with a Gaussian distributed velocity with mean 0.19 µm/s and a standard deviation of 0.038 µm/s. These values are matched to the experiments. For detailed information regarding the generic BD model, see the SI.

The results of the simulations feature the same fundamental phenomenology given by the distinct transition between the arch- and the cohesion-dominated regime (Fig. 2a–d insets), and, thus, confirm the generality of the underlying clogging phenomena (supplementary movies M7 to M9). Compared to the experiments, we find a smaller value for ${\beta }_{0}$, namely ${\beta }_{0} \approx 1.5$. This value is just above the point where cohesion overcomes the prefactor of alignment (i.e. $\beta > \gamma )$. An explanation for this mismatch might be the presence of additional interparticle forces and the particle-collision avoidance motion in the experiments, which have to be overcome by the cohesive interaction. Strikingly, the simulations qualitatively reproduce the exponential distribution of the burst size (Fig. 2f inset), the power-law behavior of $P(t \ge \tau )$ (Fig. 3a inset) and the constant particle flow rate for $\beta < {\beta }_{0}$ followed by the sudden drop of ${R}_{\text{flow}}$ at ${\beta }_{0}$ (Fig. 4a inset) remarkably well. For sufficient $d$ (and $\theta )$, the simulations also confirm that the droplets formed at the aperture seem to eventually detach even for large $\beta$ (Fig. 2c,d inset and supplementary movie M9), which coincides with the finite flow rate found for $\beta /{\beta }_{0} >1$(Fig. 4a).

The exponents $\alpha$ of the simulations with $d=2.3 \sigma$ and $\theta =90^\circ$ are in the same value range compared to the corresponding experiments. The resulting $\alpha$ of both the simulations and the experiments start at $\alpha >3$ and fall in the vicinity of $\alpha \approx 2$ for increasing $\beta$. Note that for intermediate and large $\beta$ values, the numerical data of some parameter combinations indicate kinks or multiple power-law sections in the progression of the CDFs (for example, Fig. 3a, $\beta /{\beta }_{0}=1.75$). This might result from the combination of the different clogging mechanisms (arch and droplet formation) acting on different time scales. It is likely that we are not able to resolve this in the experiments, which could explain deviations between the numerical and experimental exponents. While approaching $\alpha \approx 2,$ the numerically determined exponents for a system with $d=2.3 \sigma$ and $\theta =90^\circ$ stay just above $\alpha =2$ (Fig. 3a inset).

The general behavior of the flow rate of the simulations with $\theta = 90^\circ$ are in excellent qualitative agreement with the experimental results (especially for $d=2.3 \sigma$). Keeping the generic nature of the minimal BD model in mind, we also find a rather good quantitative accordance regarding ${R}_{\text{flow}}(\beta = 0)$, as the particle flow rates differ only by the factors 0.62, 1.02 and 1.53 for $d = 1.3 \sigma$, $d=2.3 \sigma$ and $d=3.9 \sigma$, respectively. However, a distinct difference becomes apparent by taking the results for $\theta = 12{0}^{\circ }$ of the experiments (Fig. 4b) and the simulations (Fig. 4b) into account: In the experiments, the flow rate is independent of $\theta ,$ but it is strongly dependent on $\theta$ in the simulations. This phenomenological deviation is presumably a direct result of the most apparent difference between the simulations and the experiments, namely the particle dynamics inside the bottleneck.

Compared with the experimental situation shown in Fig. 5a, the corresponding simulated trajectories appear much more static (Fig. 5a inset). This is also in accordance with the fact that p($\Delta$) is narrowly distributed around approximately 0 for all $\beta$, indicating a solid-like behavior (Fig. 5b inset). The rather static nature of the simulated APs leads to emerging crystallites at the bottleneck barriers, which can be commensurable or non-commensurable depending on $\theta$. Naturally, this influences the flow rate. The $\theta$-dependence of the simulation also leads to larger quantitative deviations between the experimentally and numerically calculated $\alpha$ for other angles than $\theta = 90^\circ$. For $\theta =120^\circ$ and larger $\beta ,$ a comparison with experiments is not possible as our simulation time scale (approximately $1{0}^{5}$ s) is not sufficient to determine a meaningful value for $\alpha$ or ${R}_{\text{flow}}$ due to the formation of a single crystallite clogging the flow. The insufficient simulation time scale is also the reason for the non-vanishing flow rate for $\beta > {\beta }_{0}$ (Fig. 4b, $c=1, \theta =120^\circ$).

We assign the static trajectories of the simulations to several simplifications made in the numerical model. Firstly, the already mentioned finite orientational alignment rate of the experimental APs leads to the above explained collision-avoidance mechanism, which is not fully covered in the simulation, as the AP in the numerical simulations can rotate instantaneous. This would explain the missing $\beta$-dependence in p($\Delta$) of the simulations (see Fig. 5b inset). Secondly, it is known that hydrodynamic interactions can increase the particle mobility even at low Reynolds numbers, as soon as two or APs are close by^39–42^. A further observation is the appearance of convection-like eddies in the bottleneck in the experiment (Fig. 5a) compared to less complex flow patterns in the simulations (Fig. 5a inset). These can be explained by inhomogeneities in the barrier roughness as shown in^43^. Note that integrating the responsible effects into simulations would be at the cost of a highly increased computational effort.

To test if an unexpectedly strong relative AP motion can be responsible for dissimilarities in the particle flow rate between the simulations and the experiments, we artificially increase the strength of the thermal fluctuations by a factor $c\ge 1$ in the simulations. This is done via the introduction of an effective temperature ${T}_{\text{eff}}=c T.$ See the SI for more information. The elevation of the temperature compared to the baseline setup is motivated by the strong relative movement of the APs in the experiments which is the result of phenomena like the finite orientational alignment rate, hydrodynamic interactions and the formation of eddies.

Figure 4b shows the simulated flow rates for a bottleneck with $d = 2.3 \sigma$ and the angles $\theta = 90^\circ$ and $\theta = 12{0}^{\circ }$ for different effective temperatures. Indeed, for $c = 3.5$, the $\theta$-dependence is (partially) resolved, since ${R}_{flow}$ is distinctly increased for $\theta = 12{0}^{\circ }$ and small $\beta$. Interestingly, the introduction of the effective temperature also reduces the quantitative differences of ${R}_{\text{flow}}$ between the experiments and the simulations for $\beta > {\beta }_{0}$. Here, a factor of $c=2.5$ reduces the deviation of ${R}_{\text{flow}}(\beta = 2.5{\beta }_{0} )$ for $\theta =90^\circ$ from 47.5% to 11.1%. This solidifies the fact that the strong relative particle motion supports the finite flow rate for large $\beta$. Note that the effective temperature also distinctly broadens the p($\Delta$) (dashed curve, Fig. 5b inset), even though the maximum of the distribution remains at $\Delta \approx 0$. Therefore, the introduction of an increased effective temperature ${T}_{\text{eff}}$ seems to be a promising approach to describe the flow of cohesive APs through bottlenecks.

## Conclusion

With experiments and numerical simulations, we have studied how a group of APs is able to move through a geometric constriction in dependence of cohesive interactions between the APs. Similar to granular systems, we found an exponential distribution of burst-sizes and power-law-distributed clogging durations for different values of the geometrical properties of the bottleneck and different values of the particle cohesion strength $\beta$. Upon increasing cohesion between APs, we observe a transition from an arch-dominated clogging regime to a cohesion-dominated regime. Strikingly, the flow-rate only weakly depends on $\beta$ in the arch-dominated regime which suggests that cohesion must not necessarily have a large detrimental effect on the group’s efficiency passing through geometric constructions or pores. Such behavior has been explained by “slippery” particle bonds which avoids the formation of a rigid particle network and thus prevents clogging. Even though the motion of APs is considerably more complex compared to Brownian or granular systems, a theoretical description using a minimal model with the use of an effective temperature provides good agreement with our experimental data. This suggests generic features of the intermittent flow of particles through bottlenecks independent on the specific nature of the particles which may even allow applications to living systems.

## Supplementary Information

Supplementary Video 1.

Supplementary Video 2.

Supplementary Video 3.

Supplementary Video 4.

Supplementary Video 5.

Supplementary Video 6.

Supplementary Video 7.

Supplementary Video 8.

Supplementary Video 9.

Supplementary Information.

## Data Availability

The datasets generated and/or analysed during the current study are available from the corresponding author on reasonable request.
