# Supplementary material for: Role of cohesion in the flow of active particles through bottlenecks
Source: Sci Rep. 2022 Jul 7;12:11525. doi: 10.1038/s41598-022-15577-w (PMC9262925; doi:10.1038/s41598-022-15577-w)
Supplement: Supplementary file 10 — Supplementary Information. [file 41598_2022_15577_MOESM10_ESM.pdf]

Supporting Information to Role of cohesion in the Flow of active particles through bottlenecks

Timo Knippenberg [a], Anton Lüders [a], Celia Lozano [b], Peter Nielaba [a], and Clemens Bechinger [a]

[a] Fachbereich Physik, Universität Konstanz, 78457 Konstanz, Germany
[b] Bosonit, AI Department, 26006, La Rioja, Spain

Experimental details. The APs are fabricated from silica spheres ($\sigma = 6.16$ µm diameter, microParticles GmbH). For coating, a monolayer of these particles is prepared on a microscope slide and an 80 nm Carbon-layer is sputtered on top. The particles are suspended in a binary mixture of water and PnP (0.4 mass fraction). This is a critical binary mixture with a lower critical point at 31.9°C [2] and a viscosity of 0.004 Pa s. The suspension is given in a glass capillary of 200 µm height, put in the experimental setup and kept at 26°C. Heating up the carbon caps of the particles causes a local demixture of the surrounding fluid. This creates a PnP-rich phase around the hydrophobic cap. The anisotropic body force inside this phase pushes the particle [2], the resulting active motion is with the uncapped side in front. Activity is induced by aiming a 523 nm laser spot of roughly 3.5 mW and a beamwaist of 4 µm at the particles. Changing the beam-position is achieved by using an AOD in combination with a 4f optical setup. The particles in the sample are repeatedly scanned with 0.1 MHz. Particle

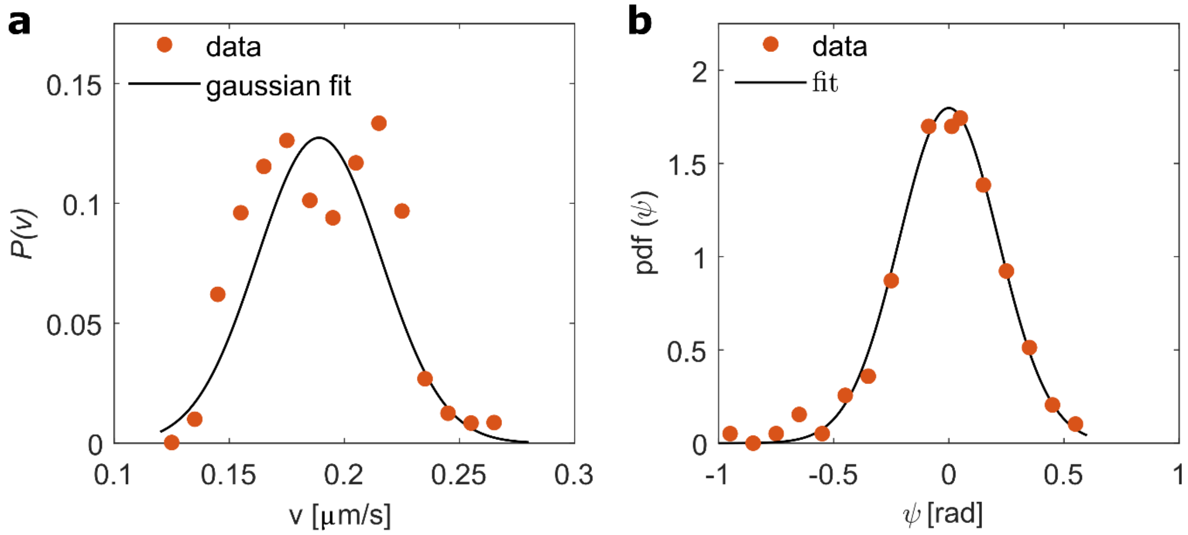

Fig. S 1 Particle Characterisation | (a) Velocity distribution obtained by analyzing the trajectory of APs acting as active Brownian particles without further interaction rule. The data is fitted with a Gaussian distribution with mean $\langle v \rangle = 0.19$ µm/s and standard deviation $\Lambda = 0.038$ µm/s. (b) Angular displacement of APs being steered to move in a straight line, obtained after 127 s. The data is fitted with $8.3 \cdot 10^{-10}$ exp$(21.5 \cdot cos\ (\psi))$.

positions are updated with 4 Hz, the detection is implemented via microscope video imaging and directly linked to the AOD in a feedback loop. Changing the direction of motion is achieved by offsetting the centre of the laserspot 2 µm. Inhomogeneous demixing will then lead to a movement in the opposite direction, as a light gradient leads to an emerging torque which anti aligns the particle orientation to the gradient [3]. As a result, the active particles are moving with a velocity which is roughly Gaussian distributed around 0.19 µm/s and a standard deviation of 0.04 µm/s in the ensemble (see Fig. S1 a). The angular velocity of particle rotation due to the steering torque is estimated as $\omega \approx 1.5°/s$. The estimation is done as explained in [3], by fitting the angular displacement ψ at straight steering for $127\,s$ to a cosine potential yielding the

steering torque M and thus the maximum angular velocity via $\omega = \frac{D_R}{k_B T} M$. Here, D_R is the rotational diffusion constant.

Detailed experimental results. The detailed values of experimentally obtained flow rates R_{flow} , power-law exponents of the clogging durations α and onsets of power-law behavior τ_0 in various situations are given in table T1 (see at the end of document) and also in figure S2. Each datapoint consists of 10 measurements of 3 h duration respectively. Particle number N may vary for each measurement between 150 and 200, but in no case all particles have passed the bottleneck during measurement time, such that the number of APs is assumed to have only mere influence. The uncertainties of R_{flow} correspond to the standard deviation of the mean of the 10 underlying measurements. For details on the determination of uncertainties of α, see section "Determination of error bars for α".

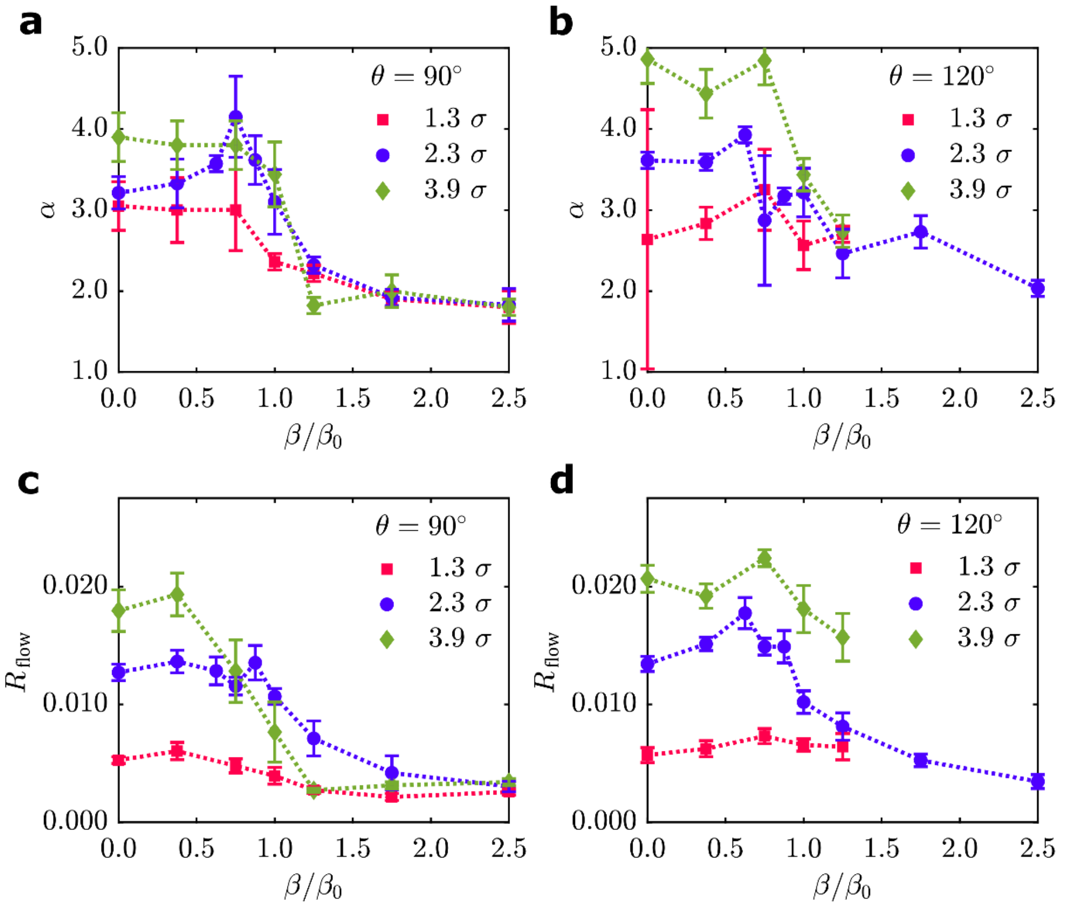

Fig. S 2 Measurement results | Measurement results of table T1 for various bottlenecks. Exponents of the power-law-decay of clogging durations in a bottleneck with (a) $\theta = 90°$ and (b) $\theta = 120°$ and different d, which are indicated by different colours. Particle flow rate in a bottleneck with (c) $\theta = 90°$ and (d) $\theta = 120°$ and different d. The error bars in (a) and (b) are determined as described in the SI. The error bars in (c) and (d) are the standard deviation of mean of the 10 single measurements.

Determination of error bars for α Since the data of all 10 measurements per datapoint is needed to have enough statistics to determine α, it is not possible to calculate the standard deviation of the mean to obtain an uncertainty interval. To circumvent this problem, we

calculate the variance σ_y of squared deviations between the CDF data points in log-log space $y_i := \log(P(t \geq \tau_i))$ and the corresponding linear fit as

$$\sigma_y = \sqrt{\frac{1}{n-2}\sum_{i=1}^{n}\left(y_i - (A + B\,log(\tau_i))\right)^2} \qquad \text{(S1)}$$

where n is the number of datapoints and A and B are the fit parameters. Note, that we only take data points $\tau_i \geq \tau_0$ into account here, to make sure we are in the linear part. For a least square fit of this linear function, it is,

$$B = \frac{n \cdot \sum_i log(\tau_i)\, y_i - (\sum_i log(\tau_i)) \cdot (\sum_i y_i)}{n \cdot (\sum_i log(\tau_i)^2) - (\sum_i log(\tau_i))^2} \qquad \text{(S2)}$$

can be found by minimizing σ_y under A and B.
We take $B = \alpha$ as this is the resulting slope independently determined with the procedure described in [4]. With geometric propagation of uncertainty, it follows then for the uncertainty interval of α:

$$u(\alpha) = \sqrt{\frac{n \cdot \sigma_y^2}{n(\sum_i log(\tau_i)^2) - (\sum_i log(\tau_i))^2}} \qquad \text{(S3)}$$

Note that this estimation does not take the uncertainty in the determination of τ_0 into account. Thus, this estimation represents a minimal uncertainty under the assumption that the CDF data indeed have a power-law distributed interval and that the size and position of this interval has been detected correctly.

Particle dynamics measurements To measure the particle dynamics, we confined approximately 150 particles in a rectangular confinement and measured their positions for 8000 s. The resulting probability density functions p(Δ) are plotted in Fig. S3 a for different β/β_0. Corresponding simulation data are shown in Fig. S3 b.

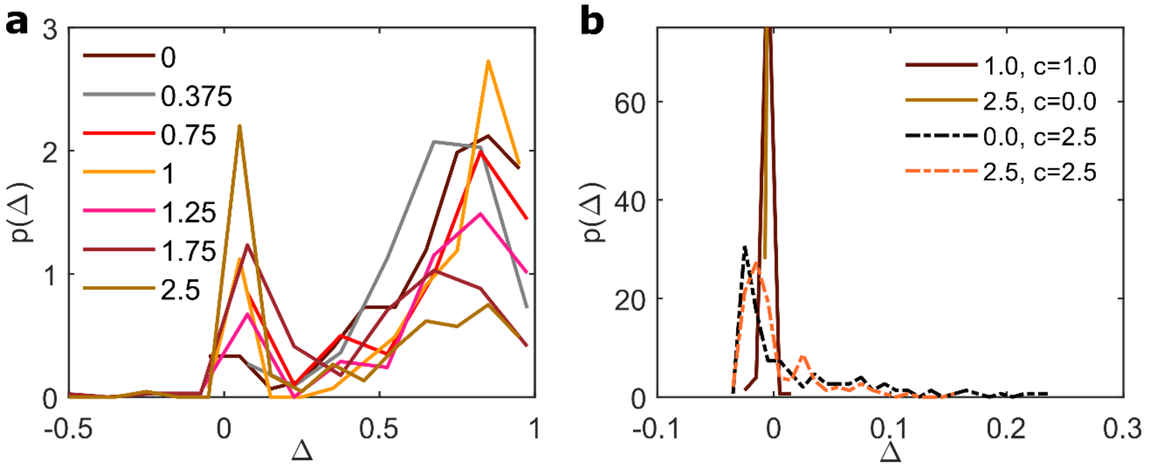

Fig. S 3 Position dynamics | (a) experimentally determined probability density functions p(Δ) for different values of β/β_0 indicated by colours. (b) Simulated p(Δ) for different values of β/β_0 and c.

Simulation details In addition to the experiments, we corroborate our experimental results by performing computer simulations. The following sections present the utilised algorithm and the methods in detail.

Brownian dynamics algorithm. We perform conventional 2D Brownian dynamics simulations [5] without hydrodynamic interactions. To integrate the overdamped motion of the centre position r_i of an AP i, the relation

$$\vec{r_i}(t+\delta t)=\vec{r_i}(t)+\vec{u_i}(t)\delta t+\frac{D}{k_{\mathrm{B}}T}\vec{F_i}(t)\delta t+\sqrt{2D\delta t}\,\vec{R_i} \tag{S4}$$

is used, where D is the diffusion coefficient of a sphere, T is the temperature, k_{B} is the Boltzmann constant, $\vec{F_i}$ is the force resulting from the interaction with the geometric constriction, δt is the length of a simulation step and $\vec{R_i}$ is a vector of standard normally distributed random numbers with $\langle\vec{R_i}(t)\rangle = 0$, $\langle R_i{}^k(t)R_j{}^l(t)\rangle = \delta_{ij}\delta_{kl}$ and $k,l \in \{x,y\}$. The vector $\vec{u_i}(t)$ is used to model the velocities of the i-th AP parallel to its orientation. In our simulations, the absolute value $v_i(t)$ of the velocity $\vec{u_i}(t)$ is a Gaussian random number with $\langle v_i(t)\rangle = 66.5\,\sigma/\tau_D$ and a standard deviation of $13.3\,\sigma/\tau_D$, where σ is the diameter of the spheres and $\tau_D = \sigma^2/D$ is the Brownian time. These values are matched to the velocity distribution of the APs of the experiments (see Fig. S1a).

The orientation angle ϑ_i of the particle i is updated according to the discrete equation

$$\vartheta_i(t+\delta t) = \vartheta_i(t) + \frac{D_{\mathrm{R}}}{k_{\mathrm{B}}T}M_i(t)\,\delta t + \sqrt{2D_{\mathrm{R}}\delta t}R_{i,\vartheta}, \tag{S5}$$

where $M_i(t)$ is the effective torque which enforces the interaction rules, $D_{\mathrm{R}} = 3/\tau_D$ is the rotational diffusion coefficient of a sphere and $R_{i,\vartheta}$ is a standard normally distributed random number.

At every simulation step, the effective torque $M_i(t)$ is calculated by

$$M_i(t) = \frac{k_{\mathrm{B}}T}{D_{\mathrm{R}}\,\delta t}[\varphi_i(t) - \vartheta_i(t)] \tag{S6}$$

The direction angle $\varphi_i(t) = \arg[\hat{\vec{u_i}}(t)]$ corresponds to the direction of motion $\hat{\vec{u_i}}(t)$ that is predetermined via the interaction rules. Note that $\hat{\vec{u_i}}(t)$ is kept constant for a time interval of length $\Delta t = \tau_D\,/\,8624$. The length Δt is matched to the actual time between two consecutive updates of the direction of motion in the experiments, which is limited by the finite sample rate (see section “Experimental details”). The effective torque corresponds to an instantaneous particle reorientation with Brownian noise.

Effective Temperature model. To study the impact of an increased relative movement of the particles inside the bottleneck, we numerically analyze systems with an elevated temperature compared to the baseline setup. For this, the diffusion constants and the temperature are artificially scaled by a factor $c \geq 1$, by replacing the corresponding quantities in all equations by the expressions

$$\begin{aligned}\bar{D} &= c\,D,\\ \bar{D}_{\mathrm{R}} &= c\,D_{\mathrm{R}},\\ T_{\mathrm{eff}} &= c\,T.\end{aligned} \tag{S7}$$

Essentially, this reduces the quotient of the propulsion strength and the strength of the thermal fluctuations. Because we utilise these simulations as a simplistic toy model to encompass some of the complex experimental phenomenology that arise, for example, due to hydrodynamic phenomena, we refer to the simulations with an elevated temperature as systems with an effective temperature of $T_{\mathrm{eff}} = c\,T$.

Interactions with the geometric constriction. To model the bottleneck, we utilise stationary Kihara spherocylinders as barriers. In the Kihara model [6], the shortest distance r between the centre of an AP and the line segment inside of a spherocylinder is used to compute the corresponding pair interaction. The shortest distance r can be calculated by the algorithm of [5], where the APs are treated as spherocylinders with aspect ratio $p = 1$. For more information regarding the AP-spherocylinder interaction, see [7,8].
The pair potential used for steric interactions between the APs and the barriers in our study is given by the Weeks-Chandler-Andersen (WCA) potential [9]

$$\mathrm{V}(r) = \begin{cases} 4\mathrm{E}_{\mathrm{BN}}\left[\left(\frac{\sigma_{\mathrm{eff}}}{\mathrm{r}}\right)^{12} - \left(\frac{\sigma_{\mathrm{eff}}}{\mathrm{r}}\right)^{6}\right] + \mathrm{E}_{\mathrm{BN}} & , r \leq 2^{1/6}\sigma_{\mathrm{eff}} \\ 0 & , r > 2^{1/6}\sigma_{\mathrm{eff}} \end{cases} \tag{S8}$$

where E_{BN} is the interaction strength. In all simulations, we use $E_{\mathrm{BN}} = 1\ k_{\mathrm{B}}T$ for simplicity. The effective diameter for the interaction is $\sigma_{\mathrm{eff}} = \sigma/2$ which corresponds to barriers or Kihara spherocylinders with a vanishing width. Note that the resulting interaction term $\frac{D}{k_{\mathrm{B}}T}\vec{F_i}(t)\ \delta t$ is independent of the qunatitiy c of the effective temperature model.
Identically to the experiments, the spherocylinder barriers are intransparent. This means that the interaction rules for the formation of cohesive groups are not calculated for a pair of APs, if the corresponding line connecting the particle centres intersects the barriers.

Interaction rules of the simulations. The interaction rules for the formation of cohesive groups utilised in the simulations are identical to their experimental counterparts. The corresponding equations to calculate the direction of motion $\widehat{\vec{u_i}}(t)$ of an AP i can be found in the main article. The only difference is that the scaling constant ε of the short-ranged repulsion is replaced by a function $\varepsilon(r_{ij})$ that diverges quickly for distances r_{ij} smaller or equal than r_{c}. In detail, we use the expression

$$\varepsilon(r_{ij}) = 4\mathrm{E}_{\vartheta}\left[\left(\frac{\sigma}{\mathrm{R}(\mathrm{r_{ij}})}\right)^{12} - \left(\frac{\sigma}{\mathrm{R}(\mathrm{r_{ij}})}\right)^{6}\right] + (\mathrm{E}_{\vartheta} + \Omega) \tag{S9}$$

with

$$R(r) = 2^{1/6}[r - r_{\mathrm{c}} + \sigma] \tag{S10}$$

This corresponds to the functional progression of a shifted and scaled WCA potential. The constant

$$\Omega = \frac{r_{\mathrm{e}} - r_{\mathrm{c}}}{r_{\mathrm{e}} - r_{\mathrm{a}}} \tag{S11}$$

guarantees that the interaction rules are repulsive at all distances $r_{ij} < r_{\mathrm{e}}$ for a system of two particles and $\beta \in [0,4]$. Throughout all simulations, we use $E_{\vartheta} = 100$. Note that the term $\varepsilon(r_{ij})\ \frac{\vec{r_j}(t) - \vec{r_i}(t)}{r_{ij}}$ should not be confused with the gradient force of an interparticle interaction based on the WCA potential. It should rather be interpreted as a vector with a length proportional to the energy of a WCA-like pair interaction.
If a particle i does not possess any neighbours with a centre-to-centre distance $r_{ij} \leq r_0$ and $\gamma = 0$ holds, then $M_i(t) = 0$ is utilised until the next calculation of $\varphi_i(t)$ (after the time interval

of length Δt) to avoid undefined behavior. This is also the case if the interaction rules give $\widehat{\vec{u_i}}(t) = 0$.

Simulation parameters. The simulations consist of $N = 150$ APs, which are placed in a square simulation box with box length $L = 10^5\sigma$. With this box length, the particles can not reach the boundaries throughout all simulations. Altogether $7.5 \cdot 10^6$ simulation steps with a length of $\delta t = 10^{-5}\,\tau_\mathrm{D}$ are performed. For systems with a large flow rate, all 150 APs pass the bottleneck in this simulation time scale. Here, we assume that the influence of the change of the particle number inside the bottleneck on the flow dynamics is negligible in a sufficient approximation within our minimal BD model. This is reaffirmed by the excellent qualitative agreement between the experiments and the simulations. For systems with smaller flow rates, not all particles can pass the bottleneck for the chosen step number, so that the number of remaining APs is assumed to have only mere influence. We chose a length of $l = 100\,\sigma$ for the line segments of the Kihara spherocylinders barriers which form the bottleneck. For a given β, up to 300 simulations are performed to gather enough statistics. All simulations are performed in units of σ, D and $k_\mathrm{B}T$. The unit of time is the Brownian time τ_D. To convert between the units of the simulations and the experiments, we use the corresponding data of the experimental setup, σ = 6.16 µm and $D = 0.0176$ µm^2/s (measured via the mean squared displacement and in accordance with [10]). This gives $\tau_\mathrm{D} = 2156$ s. The quantities $r_0, r_\mathrm{a}, r_\mathrm{e}$ and r_c are set to the values used in the experiments.

Initial conditions and position of the bottleneck. At the start of a simulation, the APs are placed in a tilted triangular lattice (with a tilt angle of $105°$) as depicted in Fig. S4. The initial orientation angles $\vartheta_i(0)$ are chosen randomly and the lattice constant is set to r_e. The initial centre of mass position of the system is positioned at $(0{,}2.5 \cdot 10^5)$.
After $2 \cdot 10^5$ simulation steps, the stationary spherocylinders, which model the barriers of the bottleneck, are placed in the simulation box. In detail, they are positioned so that the aperture has a distance of $20\,\sigma$ to the AP with the highest $\vec{r_i}(t) \cdot \vec{e}_\mathrm{drive}$ value. The quantity $\vec{r_i}(t) \cdot \vec{e}_\mathrm{drive}$ corresponds to a positioning of the APs regarding a coordinate axis parallel to the direction of motion of the cohesive group.

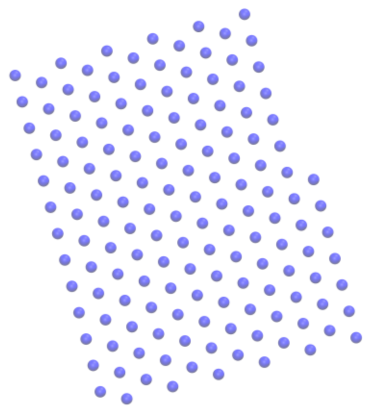

Fig. S 4 Initial conditions | Initial positions of the particles in the simulations. The APs are placed in a tilted triangular lattice with a particle distance of r_e. The initial orientations are chosen randomly. The figure was created using VMD [1].

There are two distinct parameters with which the bottleneck can be varied analogously to the experiments: Firstly, the width of the aperture d can be changed. Secondly, the angle enclosed by the two spherocylinders θ can be adjusted. Identically to the experiments, a small area behind the geometric constriction is utilised to determine the time τ between the passage of two consecutive APs. This small surface with an area of $d \cdot \sigma$ directly borders on the aperture.

Convergence of the exponent α. The total number of $7.5 \cdot 10^6$ steps per simulation corresponds to a time scale of 161700 s. This large time frame can be utilised to test possible deviations of the exponent α due to the finite observation time of our studies. In detail, the convergence of α can be analyzed by limiting the clogging times τ used to compute the CDFs at a predefined time value. This time value mimics the finite measurement time scale t_{meas} of the experiments and, thus, it is also referred to as t_{meas} for simplicity. Fig. S5 depicts the resulting exponent α in dependence on the predefined limit t_{meas} for the systems shown in Fig. 3a of the main article with $d = 2.3\,\sigma$ and $\theta = 90°$. The dashed line corresponds to the measurement time scale of the experiments of approximately 4000 s.

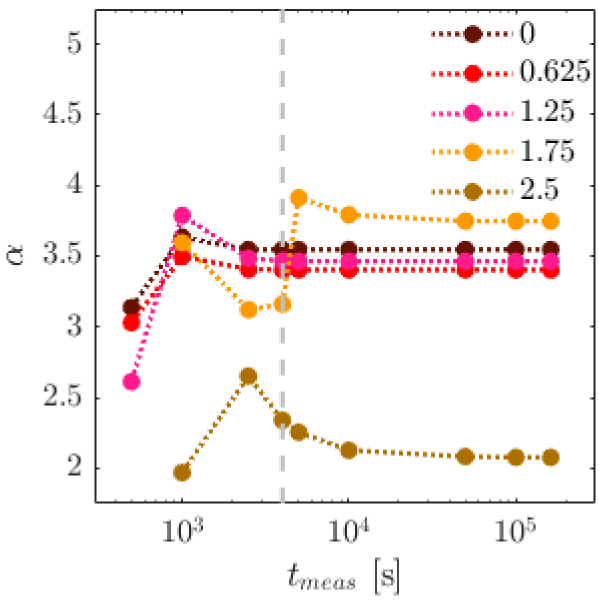

Fig. S 5 Convergence of α | Exponent α in dependence on t_{meas} for different β/β_0 values.

All studied systems (with $\theta = 90°$) easily reach a plateau in the time frame of the simulations. Therefore, the total duration of the simulations is sufficient to guarantee that the exponents of the analyzed systems reach their final values. The exponents of systems with small β are already constant at the dashed line. However, the exponents that correspond to large β converge relatively slowly compared to the experimental measurement time scale. This slow convergence could lead to imprecise experimental α values, if the general convergence behavior of the simulations and the experiments are similar.
Note that due to commensurability effects, the simulation time of 161700 s is not always sufficient at an angle of $\theta = 120°$, especially for large β. Thus, the calculations of quantities like the exponent α via simulations could lead to quantitatively and qualitatively wrong results.

Flow rate shift based on the finite measurement time scale. A rough assessment of the order of magnitude for the systematic error due to the finite measurement time scale t_{meas} of the experiments can be approximated using some simple mathematical estimations. For this, we assume that the probability density that corresponds to the experimental measurements can be roughly approximated by $p_{\mathrm{exp}}(\tau) \approx p(\tau)\,/\,q$ in its whole range of definition $[0, t_{\mathrm{meas}}]$. Here, t_{meas} is the maximum clotting time which can be observed, $p(\tau)$ is the real probability density (corresponding to an infinite observation time) and $q \propto \int_0^{t_{\mathrm{meas}}} p(\tau)\mathrm{d}\tau$ with $q \leq 1$ is a normalization (guaranteeing that $\int_0^{t_{\mathrm{meas}}} p_{\mathrm{exp}}(\tau)\mathrm{d}\tau$ leads to a probability of 1). To simplify the

notation, we use the abbreviations $[\cdot]_{\exp}$ for the average regarding $p_{\exp}(\tau)$ and $[\cdot]$ for the mean corresponding to $p(\tau)$.

The flow rate R_{flow} based on the average $\langle\tau\rangle_{\text{meas}} = [\tau]_{\exp}$ can be written in dependence on $[\tau]$ using the inequality

$$R_{\text{flow}} = \frac{1}{\langle\tau\rangle_{\text{meas}}} = \frac{1}{\int_0^{t_{\text{meas}}} \tau\, p_{\exp}(\tau)\,\mathrm{d}\tau} = \frac{q}{\int_0^{t_{\text{meas}}} \tau\, p(\tau)\,\mathrm{d}\tau} \leq \frac{1}{\int_0^{t_{\text{meas}}} \tau\, p(\tau)\,\mathrm{d}\tau} = \frac{1}{[\tau] - \int_{t_{\text{meas}}}^{\infty} \tau\, p(\tau)\,\mathrm{d}\tau}\,. \tag{S12}$$

This result can be simplified by introducing the dimensionless factor $0 \leq k < 1$ with $\int_{t_{\text{meas}}}^{\infty} \tau\, p(\tau)\,\mathrm{d}\tau = k\,[\tau]$. Note that $[\tau]$ cannot vanish in our study as two APs cannot pass the aperture simultaneously. We further assume that t_{meas} is large enough to guarantee the passage of at least two particles. With this, $k < 1$ holds.

Utilizing the factor k, we can now write

$$\frac{1}{[\tau]} \leq \mathrm{R}_{\text{flow}} = \frac{1}{[\tau]_{\exp}} \leq \frac{1}{[\tau]} + \frac{1}{[\tau]}\left(\frac{1}{1-\mathrm{k}} - 1\right) = \frac{1}{[\tau]} + \frac{\zeta}{[\tau]} \tag{S13}$$

to study the maximum discrepancy with the upwards shift function $\zeta = \frac{1}{1-k} - 1 > 0$. Here, we utilise that $[\tau] \geq [\tau]_{\exp}$ which is, at least, the case for $q \to 1$ (i.e., a rather good particle flow).

This should be valid for a test system with $d = 2.3\,\sigma$ and $\theta = 90°$. For a system with large flow rates, the factor k vanishes and R_{flow} is exact. However, if the particles pass the bottleneck only slowly, k converges to 1 and the discrepancy between R_{flow} and the exact rate $1/[\tau]$ can, in theory, diverge. We now assume that the flow rate is always equal to the upper limit of inequality (S13). This means,

$$\mathrm{R}_{\text{flow}} = \frac{1}{[\tau]} + \frac{\zeta}{[\tau]} \tag{S14}$$

In general, the exact value of k is dependent on θ, d and β. While it is not possible to compute the correct value of k or ζ, the simulations can be used to estimate a rough approximation using the relation

$$k = \frac{\int_{t_{\text{meas}}}^{\infty} \tau\, p(\tau)\,\mathrm{d}\tau}{[\tau]} \approx \frac{[\tau]_{\text{sim},\tau > t_{\text{meas}}}}{[\tau]_{\text{sim}}}. \tag{S15}$$

Here, $[\tau]_{\text{sim}}$ is the average regarding the probability density of the simulations (with a notably larger simulation time scale compared to the time scale of the experiments) and $[\tau]_{\text{sim},\tau > t_{\text{meas}}}$ is the average of all numerically obtained clogging times larger than t_{meas} (of the experiments).

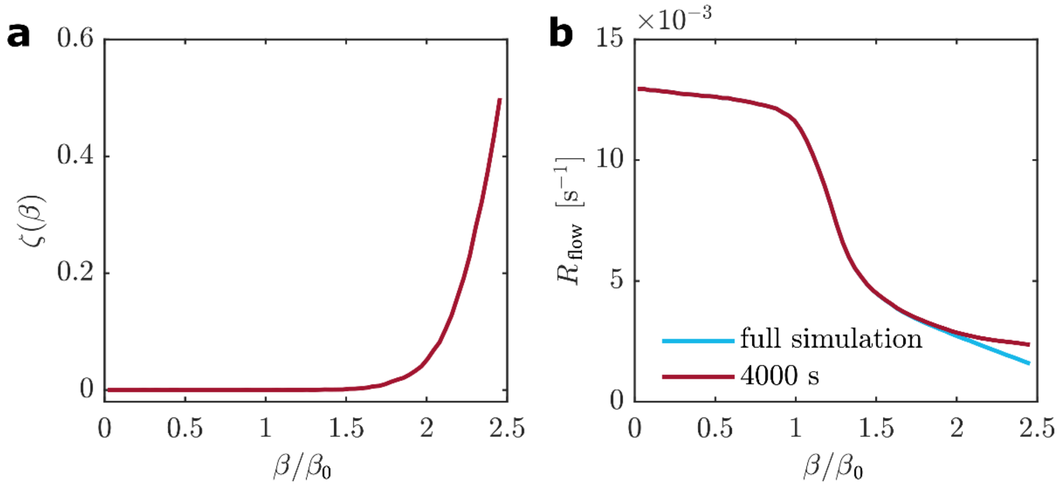

Fig. S 6 Influence of finite measurement time | (a) Upwards shift function ζ at $t_{\text{meas}} = 4000\,\text{s}$ for varying β/β_0 of a system with $d = 2.3\,\sigma$ and $\theta = 90°$. (b) Resulting simulated flow rate, evaluated after $t_{\text{meas}} = 4000\,\text{s}$ and after the full simulation duration of approximately $1.6 \cdot 10^5$ s.

Within the limit of this approximation for k, the shift function ζ can be calculated for different parameters. Fig. S6 (a) shows ζ for different β, $t_{\text{meas}} = 4000\,\text{s}$ and a bottleneck with $d = 2.3\,\sigma$ and $\theta = 90°$. The corresponding numerically calculated rate (blue) and a prediction for the experimental progression (red) based on the upwards shift function ζ are depicted in Fig.S6 (b). In the range of smaller β, the flow rate R_{flow} is larger. This results in a vanishing upwards shift function ζ. Therefore, the experimental flow rate is exact as $\langle t_{\text{tot}} \rangle = [\tau]$. For approximately $\beta > 1.75\,\beta_0$, the ζ function increases rapidly. This results in an upwards shift of the prediction flow rate compared to the numerically calculated rate. However, the discrepancy is small because $\zeta < 0.6$ holds for all analyzed β.
Strikingly, the flow rate with a finite observation time scale of 4000s is basically exact for the majority of analyzed β. This underlines the excellent suitability of R_{flow} to characterise the effectivity of how a cohesive group is able to pass a geometric obstacle.

Information on supplementary movie files

- M1.avi: Group of APs following the interaction rule with $\beta = 3.5$, particle positions were tracked from experiment and animated to show the full trajectories in lab-frame (Sample is on a comoving stage). Accelerated by a factor of 500.
- M2.avi: AP flow through bottleneck with $\theta = 90°$ and $\beta = 0$ ($\beta/\beta_0 = 0$). Video is accelerated by a factor of 200.
- M3.avi: AP flow through bottleneck with $\theta = 90°$ and $\beta = 5$ ($\beta/\beta_0 = 1.25$). Video is accelerated by a factor of 200.
- M4.avi: AP flow through bottleneck with $\theta = 90°$ and $\beta = 10$ ($\beta/\beta_0 = 2.5$). Video is accelerated by a factor of 200.
- M5.avi: AP flow through bottleneck with $\theta = 90°$ and $\beta = 4$ ($\beta/\beta_0 = 1$). Video is accelerated by a factor of 200.
- M6.avi: APs in a rectangular confinement. Interacting with $\beta = 0$ ($\beta/\beta_0 = 0$). Video is accelerated by a factor of 200.
- M7.avi: Simulation of AP flow through bottleneck with $\theta = 90°$ and $\beta = 0$ ($\beta/\beta_0 = 0$). In the video, real-time is accelerated by a factor of 650. The movie was created using VMD [1].

- M8.avi: Simulation of AP flow through bottleneck with $\theta = 90° \text{ and } \beta = 1.875$ ($\beta/\beta_0 = 1.25$). In the video, real-time is accelerated by a factor of 950. The movie was created using VMD [1] .
- M9.avi: Simulation of AP flow through bottleneck with $\theta = 90° \text{ and } \beta = 3.75$ ($\beta/\beta_0 = 2.5$). In the video, real-time is accelerated by a factor of 5100. The movie was created using VMD [1] .

Table T1 Measurement results | Resulting power-law exponents α of clogging durations, onsets of power-law behavior τ_0 and flow rates R_{flow} for bottlenecks with different θ and d at various cohesion parameters β.

θ [°]	d [σ]	β	α	τ_0 [s]	R_{flow} [1/s $\cdot 10^{-3}$]
90	1.3	0<br>1.5<br>3<br>4<br>5<br>7<br>10	3.1 ± 0.3<br>3.0 ± 0.4<br>3.0 ± 0.5<br>2.4 ± 0.1<br>2.2 ± 0.1<br>1.9 ± 0.1<br>1.8 ± 0.2	411<br>552<br>356<br>189.5<br>303<br>233.75<br>409	5.3 ± 0.2<br>6.0 ± 0.7<br>4.8 ± 0.5<br>3.9 ± 0.7<br>2.7 ± 0.2<br>2.1 ± 0.3<br>2.6 ± 0.2
90	2.3	0<br>1.5<br>2.5<br>3<br>3.5<br>4<br>5<br>7<br>10	3.2 ± 0.2<br>3.3 ± 0.3<br>3.6 ± 0.1<br>4.2 ± 0.5<br>3.6 ± 0.3<br>3.1 ± 0.4<br>2.3 ± 0.1<br>1.9 ± 0.1<br>1.8 ± 0.2	285.25<br>224.25<br>253<br>366.25<br>279.5<br>288.25<br>195.25<br>385.25<br>274.25	12.7 ± 0.6<br>13.6 ± 0.9<br>12.8 ± 1.2<br>11.6 ± 0.7<br>13.5 ± 1.5<br>10.7 ± 0.6<br>7.1 ± 1.5<br>4.2 ± 1.4<br>3.0 ± 0.4
90	3.9	0<br>1.5<br>3<br>4<br>5<br>7<br>10	3.9 ± 0.3<br>3.8 ± 0.3<br>3.8 ± 0.3<br>3.4 ± 0.4<br>1.8 ± 0.1<br>2.0 ± 0.2<br>1.8 ± 0.1	125.5<br>126.25<br>520<br>237.5<br>203<br>160.5<br>137	18.0 ± 1.8<br>19.3 ± 1.8<br>12.8 ± 2.6<br>7.6 ± 2.6<br>2.7 ± 0.1<br>3.1 ± 0.2<br>3.4 ± 0.3
120	1.3	0<br>1.5<br>3<br>4<br>5	2.6 ± 1.6<br>2.8 ± 0.2<br>3.3 ± 0.5<br>2.6 ± 0.3<br>2.7 ± 0.1	622<br>274<br>541<br>392<br>188.75	5.7 ± 0.6<br>6.2 ± 0.6<br>7.3 ± 0.6<br>6.6 ± 0.5<br>6.4 ± 1.1
120	2.3	0<br>1.5<br>2.5<br>3<br>3.5<br>4<br>5<br>7<br>10	3.6 ± 0.1<br>3.6 ± 0.1<br>3.9 ± 0.1<br>2.9 ± 0.8<br>3.2 ± 0.1<br>3.2 ± 0.3<br>2.5 ± 0.3<br>2.7 ± 0.2<br>2.0 ± 0.1	190.5<br>135.75<br>183.25<br>87.5<br>88.5<br>370<br>452<br>450<br>337	13.4 ± 0.6<br>15.1 ± 0.5<br>17.7 ± 1.3<br>14.9 ± 0.7<br>14.9 ± 1.4<br>10.2 ± 0.9<br>8.1 ± 1.2<br>5.3 ± 0.5<br>3.5 ± 0.5
120	3.9	0<br>1.5<br>3<br>4<br>5	4.9 ± 0.3<br>4.4 ± 0.3<br>4.8 ± 0.3<br>3.4 ± 0.2<br>2.7 ± 0.2	169.75<br>166<br>151.5<br>152.25<br>102	20.7 ± 1.1<br>19.2 ± 1.0<br>22.4 ± 0.7<br>18.1 ± 2.0<br>15.7 ± 2.0

References

[1] Humphrey, W., Dalke, A. & Schulten, K. VMD: Visual Molecular Dynamics. *Journal of Molecular Graphics* **14**, 33-38 (1996).

[2] Gomez-Solano, J. R. *et al.* Tuning the motility and directionality of self-propelled colloids. *Sci Rep* **7**, 14891, doi:10.1038/s41598-017-14126-0 (2017).

[3] Bauerle, T., Loffler, R. C. & Bechinger, C. Formation of stable and responsive collective states in suspensions of active colloids. *Nat Commun* **11**, 2547, doi:10.1038/s41467-020-16161-4 (2020).

[4] Clauset, A., Shalizi, C. R. & Newman, M. E. J. Power-Law Distributions in Empirical Data. *SIAM Review* **51**, 661-703, doi:10.1137/070710111 (2009).

[5] Ermak, D. L. A computer simulation of charged particles in solution. I. Technique and equilibrium properties. *The Journal of Chemical Physics* **62**, 4189-4196, doi:10.1063/1.430300 (1975).

[6] Kihara, T. Virial Coefficients and Models of Molecules in Gases. *Reviews of Modern Physics* **25**, 831-843, doi:10.1103/RevModPhys.25.831 (1953).

[7] Luders, A., Siems, U. & Nielaba, P. Dynamic ordering of driven spherocylinders in a nonequilibrium suspension of small colloidal spheres. *Phys Rev E* **99**, 022601, doi:10.1103/PhysRevE.99.022601 (2019).

[8] Luders, A., Zander, E. & Nielaba, P. Microscopic diffusion coefficients of dumbbell- and spherocylinder-shaped colloids and their application in simulations of crowded monolayers. *J Chem Phys* **155**, 104113, doi:10.1063/5.0060063 (2021).

[9] Weeks, J. D., Chandler, D. & Andersen, H. C. Role of Repulsive Forces in Determining the Equilibrium Structure of Simple Liquids. *J. Chem. Phys.* **54**, 5237 (1971).

[10] Gomez-Solano, J. R., Blokhuis, A. & Bechinger, C. Dynamics of Self-Propelled Janus Particles in Viscoelastic Fluids. *Phys Rev Lett* **116**, 138301, doi:10.1103/PhysRevLett.116.138301 (2016).
